# Lobular Capillary Hemangioma of the External Jugular Vein: A Rare Case Report

**DOI:** 10.22038/ijorl.2021.52996.2806

**Published:** 2021-07

**Authors:** Manuel Tucciarone, Luz López-Flórez, Tomas Martínez-Guirado, Rosalia Souviron-Encabo, Ricardo González-Orus Álvarez-Morujo

**Affiliations:** 1 *Department* of Otorhinolaryngology, Gregorio Marañón Hospital, Madrid, SPAIN.

**Keywords:** Hemangioma, Vascular Neoplasms, Granuloma, Pyogenic

## Abstract

**Introduction:**

Hemangiomas are benign tumors that are very common in the head and neck region. However, intravascular hemangiomas are very rare. Hemangiomas are classified as capillary, cavernous or mixed tumors according to the proliferating cells. Ultrasound, computed tomography, MR imaging and angiography are useful diagnostic tools and are generally required when planning surgical treatment. Definitive diagnosis is established by histopathological examination, differentiating hemangiomas from other vascular tumors or malignancies.

**Case Report:**

We present a rare case of capillary hemangioma protruding from the external jugular vein. In our patient, the tumor was totally removed under local anesthesia. No complications and no recurrence were observed in the following two years.

**Conclusion:**

Intravascular tumors can present as neck masses and a definitive diagnosis is made by histopathological examination. Imaging tools provide important information about anatomy, the extent of the tumor, and for surgical planning.

## Introduction

Hemangiomas are benign lesions characterized by a neoplastic proliferation of vascular endotelial cells; they are common vascular tumors in the head and neck region although their appearance as primitive intravascular proliferations is very unusual. Intraluminal hemangiomas must be differentiated from other vascular tumors and malignancies. In this article we present the case of a rare intravascular centrolobular capillary hemangioma protruding from the external jugular vein. 

## Case Report 

**Fig1:**
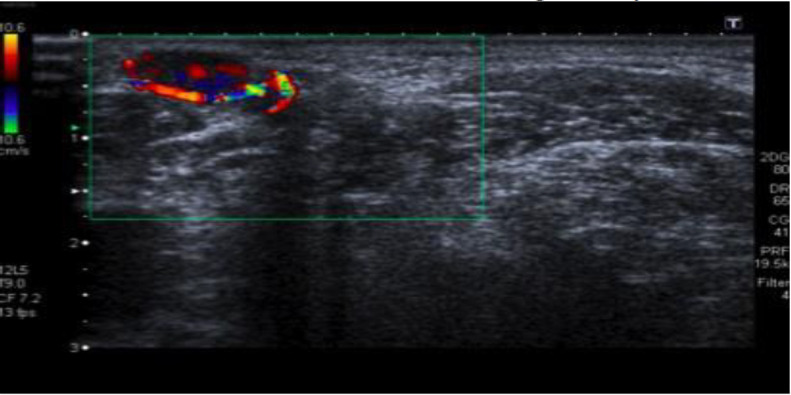
US image showing an intraluminal lesion of the left external jugular vein with intrinsic arterial and venous blood flow at the doppler scan

**Fig 2:**
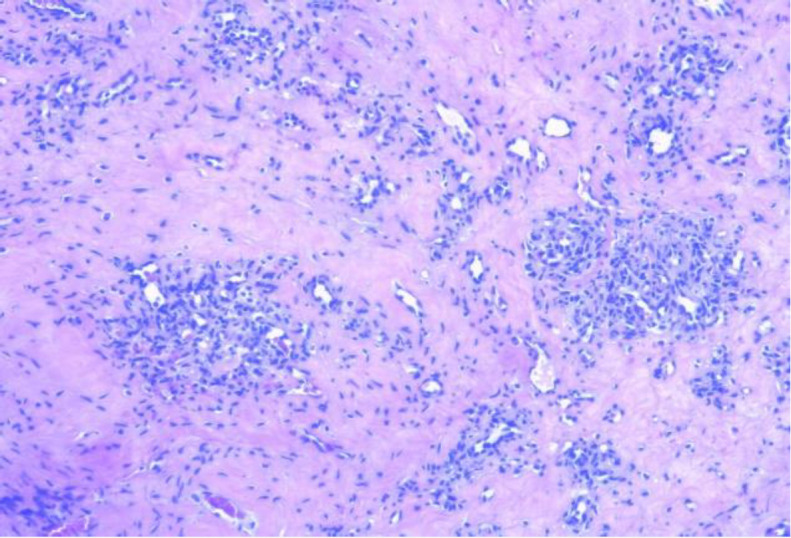
Histopathologic findings of a lobular capillary hemangioma showing that the capillaries are arranged in a lobular manner surrounded by fibromyxoid stroma

A 69-year-old woman with an unremarkable medical history came for an examination on a painless mass on the left side of her neck, which had slowly and gradually grown in size over the two previous months. She had no history of previous trauma or catheter insertions in the neck. On physical examination everything was normal except for the presence of a left-sided mobile and non-pulsating mass along the course of the external jugular vein (EJV) without any inflammatory symptoms. An echography scan was performed showing a 15 x 8 mm intraluminal lesion, with intrinsic arterial and venous blood flow on the doppler scan (Figure.1) without any vascular compromise of the vessel. A CT scan was performed and confirmed the non-occlusive intraluminal neoformation in the external jugular vein (EJV). The patient underwent surgery under local anesthesia with the total removal of the mass ligating the EJV. Histopathological examination showed images of lobulated angiomatoid proliferation of blood vessels and established the definitive diagnosis of intravenous lobular capillary hemangioma (Figure.2). No complications after surgery and no recurrence were observed over the following two years.

## Discussion

Vascular anomalies are soft tissue lesions with congenital errors in vascular development. According to the classifications of Mulliken and Glowacki (1982) (1), then revised by the International Society for the Study of Vascular Anomalies (ISSVA), vascular anomalies are divided into hemangiomas and vascular malformations (2). Hemangiomas are benign neoplastic aberrancies in which, according to the type of proliferating cells, it is possible to categorize them into three histopathologic classes: capillary, cavernous and mixed hemangiomas. Capillary hemangiomas are characterized by a profileration of capillary structures and a short clinical history; they are found predominantly in the head and neck. Cavernous ones are formed by larger vessels, have a longer clinical history and are generally discovered in the extremities and in the lower part of the body. Mixed hemangiomas have both kinds of structures and clinically are more similar to cavernous ones. Although haemangiomas are very frequent tumours, above all in children and involving skin and mucosa, primary intraluminal cases are very rare (3). Pathogenesis is still unknown; they are the result of congenital endothelial hyperplasia but some authors have described acquired conditions relating to infections, traumas and hormonal or pressure changes (4).

Intrinsic vascular malformations arising from the wall of the EJV are very rare. In English literature we found only seven articles describing intravascular tumours arising from the EJV (3,5-10). 

The first article describing a vascular intrinsic tumour of the EJV appeared in 1967 where the author wrote about the utility of venography for its diagnosis (5). Sarteschi et al. (6) wrote about the usefulness of color-coded duplex sonography for the diagnosis of an intravascular haemangioma and in the same year Ahuja et al. described the MR imaging appearance of hemangiomas of the EJV (7). External jugular venous wall’s vascular malformations have particular sonographic features, showing well-defined, ovoid, heterogeneous hypoechoic nodules with internal flow signals arising from the venous wall with endoluminal extension but no adjacent soft tissue infiltration. 

With MRI, intravascular haemangiomas typically show intermediate signal intensity on T1-weighed images, very high signal intensity on T2-weighed images, and variable enhancement after intravenous gadolinium administration. 

Although ecography, computed tomography, and MR imaging give important details about anatomy and surgical planning, the definitive diagnostic tool is the histopathological examination (8-9). 

In our patient, after ecography and a CT scan we performed an excisional biopsy under local anesthesia, ligating the EJV on both sides, and confirmed the histophatologic diagnosis and the total removal of the tumor. 

The histologic differential diagnosis of intravenous lobular capillary hemangioma includes inflammatory angiomatous nodules, intravascular papillary endothelial hyperplasia, intravenous atypical vascular proliferation, pseudo-Kaposi sarcoma, angiosarcomas, intravascular fasciitis, and an organized thrombus (10). 

We did not observe postoperative complications or recurrence after two years. In previous cases no recurrence has been reported after the total removal of the tumor. 

## Conclusion

Intravascular tumors can appear as neck masses and the definitive diagnosis is made by histopathological examination considering the importance of imaging tools to provide information about anatomy, the extent of the tumor, and for surgical planning.The authors declared no conflicts of interest with respect to the authorship and/or publication of this article. The patient has consented to the submission of the case report for submission to the journal. 
